# Decreased Jumonji Domain-Containing 3 at the Promoter Downregulates Hematopoietic Progenitor Kinase 1 Expression and Cytoactivity of T Follicular Helper Cells from Systemic Lupus Erythematosus Patients

**DOI:** 10.1155/2022/3690892

**Published:** 2022-09-28

**Authors:** Shuaihantian Luo, Yuming Xie, Junke Huang, Juan Liu, Qing Zhang

**Affiliations:** Department of Dermatology, The Second Xiangya Hospital, Central South University, Changsha, Hunan 410011, China

## Abstract

T follicular helper (Tfh) cells are overactivated in systemic lupus erythematosus (SLE) patients and contribute to excessive immunity. Hematopoietic progenitor kinase 1 (HPK1), as an inhibitor of T cells, is underexpressed in SLE Tfh cells and consequently induces autoimmunity. However, the reason for downregulation of HPK1 in SLE Tfh cells remains elusive. By combining chromatin immunoprecipitation with quantitative polymerase chain reaction assays, it was found that histone H3 lysine 27 trimethylation (H3K27me3) at the HPK1 promoter in SLE Tfh cells elevated greatly. We also confirmed jumonji domain-containing 3 (JMJD3) binding at the HPK1 promoter in SLE Tfh cells reduced profoundly. Knocking down JMJD3 in normal Tfh cells with siRNA alleviated enrichments of JMJD3, H3K4me3, and mixed-lineage leukemia (MLL) 1 at the HPK1 promoter and increased H3K27me3 number in the region. HPK1 expression was lowered, while Tfh cell proliferation activity, IL-21 and IFN*γ* secretions in the supernatants of Tfh cells, and IgG1 and IgG3 concentrations in the supernatants of Tfh-B cell cocultures all upregulated markedly. In contrast, elevating JMJD3 amount in SLE Tfh cells by JMJD3-overexpressed plasmid showed opposite effects. The abundances of H3K4me3 and MLL1 at the HPK1 promoter in SLE Tfh cells were greatly attenuated. Our results suggest that deficient JMJD3 binding at the promoter dampens HPK1 expression in SLE Tfh cells, thus making Tfh cells overactive, and ultimately results in onset of SLE.

## 1. Introduction

Systemic lupus erythematosus (SLE) is a chronic autoimmune disease, which may seriously threaten public health [1]. The basic pathogenesis of SLE is related to overactivation of T cells, which leads to overstimulation of B cells, release of inflammatory factors and autoantibodies, and autoimmune responses to multiple organs and systems [2]. However, its specific pathogenesis still remains elusive.

In recent years, a new subgroup of T cells, T follicular helper (Tfh) cells, has been discovered. They are specialized providers of T cells assisting B cells to differentiate, mature, produce high-affinity antibodies, and form germinal center (GC) [3–6]. Studies have shown that Tfh cells play a crucial role in the pathogenesis of SLE. It has been confirmed that Tfh cells account for a significantly higher proportion in peripheral blood circulation of SLE patients, and they are positively correlated with SLE disease activity index (SLEDAI), antinuclear antibody (ANA), and anti-double stranded DNA (dsDNA) antibody levels in serum and are negatively correlated with complement C3 concentration [7, 8]. The secretions of interleukin-21 (IL-21), B cell-activating factor (BAFF), interferon-*γ* (IFN*γ*), and IL-17A from Tfh cells can promote the development of SLE [9–12]. Therefore, Tfh cells have become a research hotspot in the pathogenesis of SLE.

Hematopoietic progenitor kinase 1 (HPK1), also known as mitogen-activated protein kinase kinase kinase kinase 1 (MAP4K1), is a serine/threonine protein kinase [13, 14]. It is involved in various immune responses, such as promoting the activation of c-Jun N-terminal kinase (JNK) and nuclear factor-*κ*B (NF-*κ*B), degrading inhibitor of NF-*κ*B (I*κ*B), regulating cell proliferation and apoptosis, and inhibiting T cell-mediated immune responses and T cell receptor (TCR) signaling [14–16]. Our previous study revealed that the expression of HPK1 in Tfh cells dramatically decreased in SLE patients, leading to overactivation of Tfh cells, and therefore, more IL-21, BAFF, and IFN*γ* were produced and B cells were stimulated to produce more immunoglobulin G (IgG) [17]. However, the reason for reduced expression of HPK1 in SLE Tfh cells has not been clarified.

Epigenetic mechanisms mainly involve DNA methylation changes, histone modifications, regulation of noncoding RNA, chromatin remodeling, etc. [18, 19]. Trimethylation of histone H3 lysine 27 (H3K27me3), as a marker of transcriptional inhibition, has markedly attracted scholars' attention [20–23]. According to previously reported findings, H3K27me3 could bind to the Pc protein in polycomb repressive complex 1 (PRC1) and recruit PRC1 to chromatin; PRC1 could block the binding of chromatin remodeling factors and transcription activation factors to DNA and suppress the transcription initiated by RNA polymerase II; PRC1 was also associated with histone deacetylases (HDACs) to inhibit transcription; and PRC1 and H3K27me3 could also block positive activation markers, including methylation of H3K4 [2, 22, 23]. Enhancer of zeste homolog 2 (EZH2) is known as methyltransferase of H3K27 [24], while jumonji domain-containing 3 (JMJD3) [2] and ubiquitously transcribed tetratricopeptide repeat on X chromosome (UTX) are demethylases of H3K27 [25].

Our previous study revealed that H3K27me3 level at the HPK1 promoter in CD4^+^ T cells of SLE patients significantly decreased [2]. However, the above-mentioned study only involved HPK1 at the CD4^+^ T cellular level. We guessed whether the enrichments of EZH2, JMJD3, or UTX within the HPK1 promoter region of SLE Tfh cells altered and therefore contributed to a series of epigenetic changes. All these alleviated HPK1 expression and stimulated Tfh cell overactivation. At last, overactivated Tfh cells promoted autoimmune response of SLE. This research will further uncover the epigenetic pathogenesis of SLE and is expected to provide new insights into the treatment of SLE.

## 2. Methods and Materials

### 2.1. Subjects

A total of 30 patients with SLE (SLE group; age, 28.93 ± 5.75 years old) were enrolled from the out-patient clinic and in-patient ward of the Department of Dermatology, the Second Xiangya Hospital, Central South University (Changsha, China). The data of patients are listed in Table 1. Besides, 30 healthy controls (control group; age, 27.80 ± 5.51 years) were recruited from healthy staff and graduate students of the Second Xiangya Hospital, and their data are presented in Table 2. The SLE group and control group were age and sex matched, and written informed consent was obtained from each participant before enrollment. The study was performed according to the Declaration of Helsinki, and it was approved by the Ethics Committee of the Second Xiangya Hospital, Central South University.

### 2.2. Cell Isolation

Every participant's venous peripheral blood was preserved in heparin. Peripheral blood mononuclear cells (PBMCs) were isolated from the blood by Ficoll-Hypaque density gradient centrifugation (GE Healthcare). Whereafter, naive CD4^+^ T cells were isolated by human naive CD4^+^ T cell isolation kit (Miltenyi), and B cells were collected using CD19 magnetic beads (Miltenyi), according to the protocols of the manufacturer.

### 2.3. In Vitro Differentiation of Tfh Cells

Anti-CD3 antibody (Calbiochem, 5 *μ*g/mL) was precoated in 24-well plates at 4°C overnight. Naive CD4^+^ T cells were then plated into the medium with anti-CD28 antibody (Calbiochem, 2 *μ*g/mL) and cultured with transforming growth factor *β* (TGF-*β*) (PeproTech, 5 ng/mL), IL-6 (PeproTech, 20 ng/mL), IL-12 (PeproTech, 10 ng/mL), and IL-21 (PeproTech, 20 ng/mL) in Roswell Park Memorial Institute (RPMI) 1640 medium which contained 100 U/mL penicillin G, 50 *μ*g/mL streptomycin, and 10% fatal bovine serum (FBS) at 37°C in a 5% CO_2_ atmosphere for 5 days. Subsequently, the cells were collected for further analysis.

### 2.4. Chromatin Immunoprecipitation (ChIP) Assay

The ChIP assay was performed with a ChIP kit (Millipore), as described previously [26]. Anti-H3K27me3 and anti-H3K4me3 antibodies were purchased from Millipore; anti-JMJD3, anti-MLL1, anti-MLL2, anti-MLL3, and anti-MLL4 antibodies were provided by Abcam. The immunoprecipitated DNA was purified, then subjected to quantitative polymerase chain reaction (qPCR) analysis using a Rotor-Gene 3000 thermocycler (Corbett Research Ltd.), with input DNA (total chromatin) as an endogenous control. The primers of HPK1 promoter were as follows: 5′-TGGGGAGATAGAGGTTGCAG-3′ (forward) and 5′-CGCCAGAAATCCAATGACTT-3′ (reverse).

### 2.5. RNA Isolation and cDNA Synthesis

Total RNA of Tfh cells was isolated by a RNeasy mini kit (Qiagen), according to the manufacturer's instruction. Subsequently, cDNA was synthesized from 1 *μ*g of total RNA with a miScript Reverse Transcription kit (Qiagen) and stored at −80°C.

### 2.6. QPCR

The qPCR was carried out with a Rotor-Gene 3000 thermocycler (Corbett Research Ltd.). The expression of target cDNA was measured by a SYBR Premix Ex Taq kit (TaKaRa), and *β*-actin was used as an endogenous control. A series of five dilutions from a cDNA sample were simultaneously amplified to generate a standard curve, in order to evaluate the relative amount of each cDNA sample. The primers used in this study were as follows: for HPK1, 5′-CTGCTGGAACGGAAAGAGAC-3′ (forward) and 5′-CGGACAAGCAGGAATTTGTT-3′ (reverse); for *β*-actin, 5′-CGCGAGAAGATGACCCAGAT-3′ (forward) and 5′-GCACTGTGTTGGCGTACAGG-3′ (reverse).

### 2.7. Western Blot Analysis

Tfh cells were lysed by whole-cell lysis buffer (Thermo Fisher Scientific). The lysates were centrifuged, and the proteins were quantified using a Bradford protein assay kit (Bio-Rad Laboratories Inc.). Subsequently, the proteins were separated by sodium dodecyl sulfate-polyacrylamide gel electrophoresis (SDS-PAGE) with 8% polyacrylamide gels and were transferred onto polyvinylidene difluoride (PVDF) membranes (Millipore). The membranes were blocked with Tris-buffered saline–Tween (TBST) buffer supplemented with 5% nonfat dry milk and incubated at 4°C overnight with HPK1 antibody (dilution, 1 : 100; Santa Cruz Biotechnology Inc.), JMJD3 antibody (dilution, 1 : 100; Abgent Ltd.), or *β*-actin antibody (dilution, 1 : 100; Santa Cruz Biotechnology Inc.). The blots were exposed to X-ray films, and the band densities were analyzed by the Quantity One Analysis software (Bio-Rad Laboratories Inc.).

### 2.8. Transfection

Control-siRNA and JMJD3-siRNA were designed and synthesized by Guangzhou RiboBio in China. The pcDNA3.1 blank plasmid and pcDNA3.1-JMJD3-expressing plasmid were granted by Dr. Charlie Degui Chen (Chinese Academy of Sciences). The transfections were performed with Human T cell Nucleofector kit and a Nucleofector (Amaxa), following the protocol provided by the manufacturer. The transfected Tfh cells were cultured in human T cell culture medium. After 24 h of transfection, the culture medium was added 5.0 *μ*g/mL anti-CD28 and 5.0 *μ*g/mL anti-CD3 antibodies for 48 h to stimulate Tfh cells and activate HPK1.

### 2.9. Cell Proliferation Assays

72 h after transfection, Tfh cells which had been stimulated were seeded into 96-well flat-bottomed plates (2 × 10^5^ cells every well). Whereafter, 10 *μ*L of 5 mg/mL 3-(4,5-dimethylthiazol-2-yl)-2,5-diphenyltetrazolium bromide (MTT) (Roche) was added into each well. After 4 h, these plates were centrifuged, and the supernatants were removed. The Tfh cells were subsequently dissolved in 100 *μ*L dimethyl sulfoxide (DMSO) every well at room temperature. The absorbance at 570 nm was read by an ELx800 Absorbance Microplate Reader (Bio-Tek).

### 2.10. Tfh-B Cell Costimulation Assays

After isolation, peripheral B cells were cultured in RPMI 1640 medium containing 100 U/mL penicillin G, 50 *μ*g/mL streptomycin, and 10% FBS. 72 h after transfection, Tfh cells and autologous B cells were cocultured in 96-well U-bottom plates at 37°C with 5% CO_2_ atmosphere, and the ratio of Tfh cells to B cells was 1 : 10. 50 *μ*L medium was supplemented in every well on day 4, and the supernatants were collected on day 8 to examine IgM, IgG1, IgG2, and IgG3 concentrations.

### 2.11. Enzyme-Linked Immunosorbent Assay (ELISA)

The productions of IL-21, BAFF, IFN*γ*, and IL-17A in the supernatants of stimulated Tfh cells and the secretions of IgM, IgG1, IgG2, and IgG3 in the supernatants of Tfh-B cell cocultures were measured with corresponding quantification ELISA kits (Abcam), following the instructions provided by the manufacturer. The optical density (OD) values were read at 450 nm with an ELx800 Absorbance Microplate Reader (Bio-Tek).

### 2.12. Statistical Analysis

Data were expressed as mean ± standard deviation (SD). The difference between SLE patients and healthy controls was compared with two-sample *t* test, and the results of different transfections were compared by paired-sample *t* test. The data from every control-siRNA group member were set as 1, and the multiple of every JMJD3-siRNA group member relative to its homologous control-siRNA group member was calculated. The results of blank plasmid group and JMJD3-expressing plasmid group were dealt with in the same way. The strength of correlation was analyzed using Pearson's correlation coefficient. *P* < 0.05 was considered statistically significant. The statistical analysis was performed by the SPSS 25.0 software (SPSS Inc.).

## 3. Results

### 3.1. H3K27me3 Expression Increased at the HPK1 Promoter in Tfh Cells of SLE Patients

**Figure 1:**
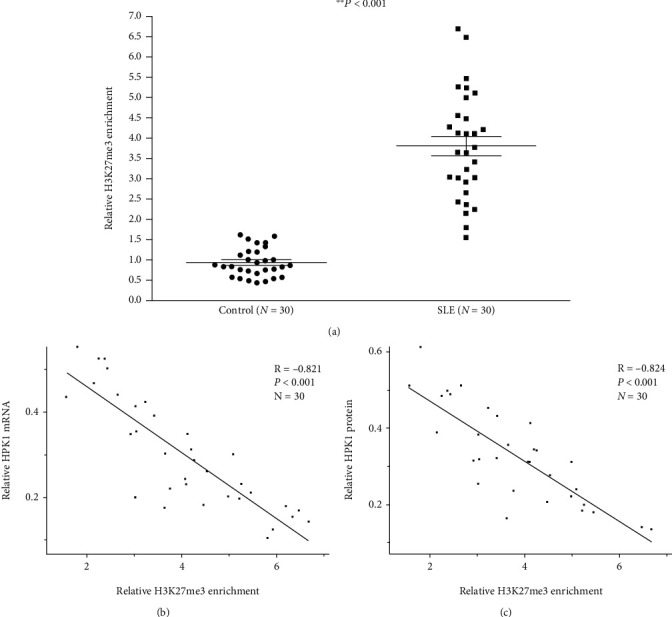
H3K27me3 enrichment at the HPK1 promoter in healthy and SLE Tfh cells. (a) Relative H3K27me3 enrichment at the HPK1 promoter in healthy and SLE Tfh cells was measured by combining ChIP and qPCR assays. Results were normalized to input DNA (total chromatin). (b, c) The correlations between HPK1 mRNA level (b), protein level (c), and H3K27me3 enrichment in SLE Tfh cells.

We combined ChIP with qPCR assays to detect the H3K27me3 expression at the HPK1 promoter region in Tfh cells from SLE and control groups. The results confirmed that the H3K27me3 enrichment at the HPK1 promoter region in SLE Tfh cells was significantly higher than that in the control group (Figure 1(a)).

In order to verify the correlation between H3K27me3 abundance and HPK1 expression, we detected HPK1 mRNA and protein levels in Tfh cells from the SLE group by qPCR and western blot analysis, respectively. The results revealed that H3K27me3 number in the promoter region was significantly negatively correlated with mRNA (Figure 1(b)) and protein (Figure 1(c)) levels of HPK1.

### 3.2. JMJD3 Binding Decreased at the HPK1 Promoter in Tfh Cells of SLE Patients

**Figure 2:**
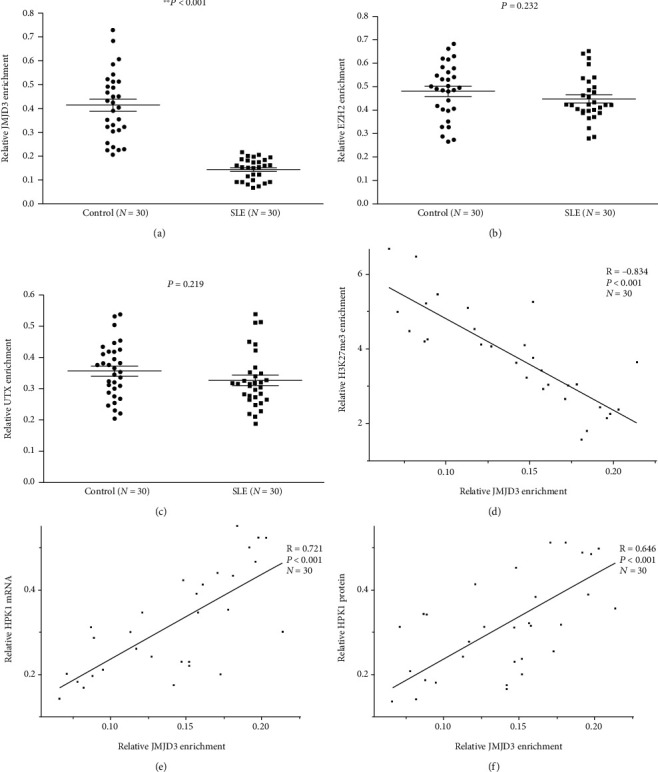
JMJD3, EZH2, and UTX bindings at the HPK1 promoter in healthy and SLE Tfh cells. (a–c) Relative levels of JMJD3 (a), EZH2 (b), and UTX (c) binding within the HPK1 promoter region in healthy and SLE Tfh cells were analyzed by combining ChIP and qPCR assays. Results were normalized to input DNA (total chromatin). (d–f) The correlations between H3K27me3 enrichment (d), HPK1 mRNA level (e), protein level (f), and JMJD3 binding in SLE Tfh cells.

In order to explore the cause of the increased H3K27me3 enrichment at the HPK1 promoter in SLE Tfh cells, the levels of EZH2, JMJD3, and UTX at the HPK1 promoter in Tfh cells from the SLE and control groups were measured by combining ChIP and qPCR assays. It was found that JMJD3 binding in the SLE group was greatly lower than that in the control group (Figure 2(a)), and there was no obvious difference in the quantities of EZH2 (Figure 2(b)) and UTX (Figure 2(c)) between the two groups. It was also revealed that the JMJD3 binding was negatively correlated with H3K27me3 enrichment at the HPK1 promoter in Tfh cells of the SLE group (Figure 2(d)), while it was positively correlated with the mRNA (Figure 2(e)) and protein (Figure 2(f)) levels of HPK1.

### 3.3. Inhibited Expression of JMJD3 in Normal Tfh Cells Reduced HPK1 Abundance, Downmodulated H3K4me3 and MLL1 Levels in the HPK1 Promoter Region, and Enhanced Tfh Cell Activity

**Figure 3:**
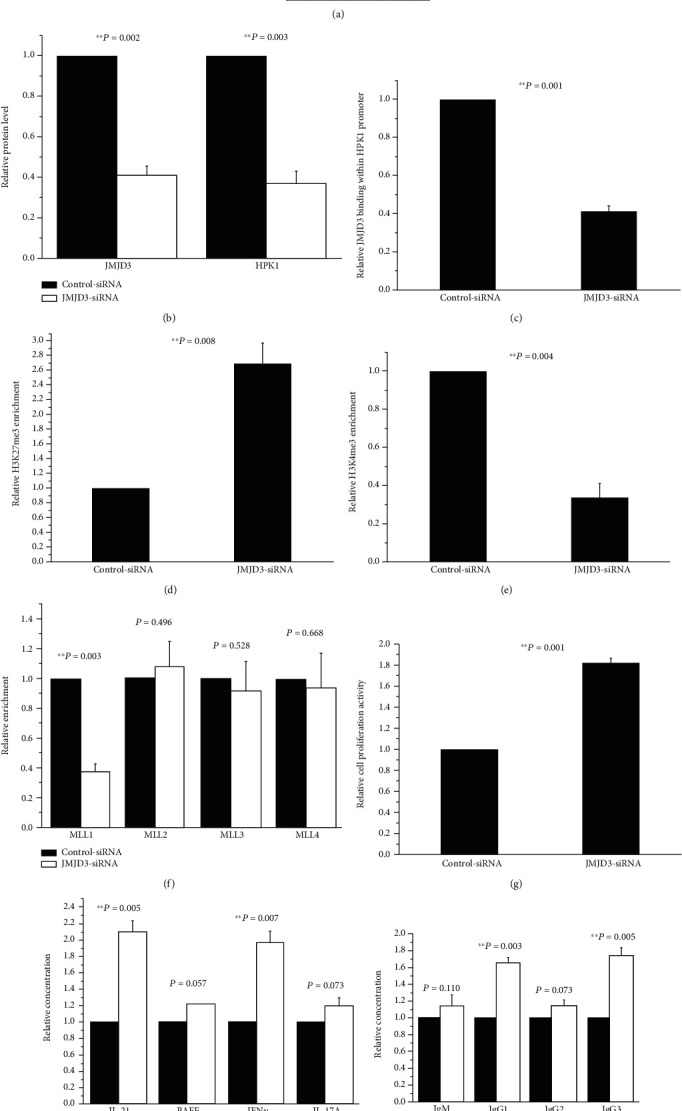
Effects of JMJD3 downregulation on Tfh cells from 3 normal controls. (a, b) Relative JMJD3 and HPK1 protein levels in normal Tfh cells transfected with control-siRNA or JMJD3-siRNA were measured by western blot analysis after stimulation. *β*-Actin was used as endogenous control. (c–f) Relative JMJD3 (c), H3K27me3 (d), H3K4me3 (e), MLL1, MLL2, MLL3, and MLL4 (f) enrichments at the HPK1 promoter in normal Tfh cells transfected with control-siRNA or JMJD3-siRNA were quantified by combining ChIP and qPCR assays after stimulation. Results were normalized to input DNA (total chromatin). (g) The proliferation activities of normal Tfh cells transfected with control-siRNA or JMJD3-siRNA were assessed by MTT assays after stimulation. (h) The relative concentrations of IL-21, BAFF, IFN*γ*, and IL-17A in the supernatants of normal Tfh cells transfected with control-siRNA or JMJD3-siRNA were examined using ELISA after stimulation. (i) After stimulation, some normal Tfh cells transfected with control-siRNA or JMJD3-siRNA were cocultured with autologous B cells for 8 days, whereafter the concentrations of IgM, IgG1, IgG2, and IgG3 in the supernatants were detected by ELISA. The data from every control-siRNA group member were set as 1, and the multiple of every JMJD3-siRNA group member relative to its homologous control-siRNA group member was calculated.

In order to confirm that the decreased JMJD3 binding at the HPK1 promoter is the cause of HPK1 underexpression in Tfh cells of SLE patients, we first transfected control-siRNA or JMJD3-siRNA into the Tfh cells from three healthy donors. After 72 h, we found that JMJD3 and HPK1 protein levels in the JMJD3-siRNA group were noticeably reduced (Figures 3(a) and 3(b)). Meanwhile, JMJD3 binding at HPK1 promoter significantly lowered (Figure 3(c)), while H3K27me3 enrichment remarkably increased (Figure 3(d)).

Studies have confirmed that H3K27 and H3K4 methylations are mutually exclusive [22]. It has also been found that UTX not only demethylates H3K27 but also recruits the MLL family of H3K4 methyltransferases, resulting in increased H3K4 methylation [27–29]. However, the relationship between JMJD3 and H3K4 methylation has not been studied. The transfected Tfh cells were assessed in the present study, and it was found that H3K4me3 proportion at the HPK1 promoter in the JMJD3-siRNA group sharply diminished (Figure 3(e)). The MLL1 binding in the region was also markedly reduced (Figure 3(f)), while the levels of MLL2, MLL3, and MLL4 did not statistically change (Figure 3(f)).

We further analyzed the influences of JMJD3 on Tfh cell activity. After 72 h of transfection, the proliferation activities of Tfh cells were assessed by MTT assays, and the quantities of IL-21, BAFF, IFN*γ*, and IL-17A in the supernatants of Tfh cells were examined by ELISA. Another part of Tfh cells transfected with siRNA was cocultured with autologous B cells for 8 days, whereafter the abundances of IgM, IgG1, IgG2, and IgG3 in the supernatants of cocultures were analyzed using ELISA. Compared to the control group, the proliferation activity of the JMJD3-siRNA group upregulated markedly (Figure 3(g)), the amounts of IL-21 and IFN*γ* also increased significantly, but BAFF and IL-17A did not alter profoundly (Figure 3(h)). And after coculturing, IgG1 and IgG3 oversecreted in the JMJD3-siRNA group, while there were no obvious differences in IgM and IgG2 levels between these two groups (Figure 3(i)).

### 3.4. Upregulation of JMJD3 in SLE Tfh Cells Elevated HPK1 Expression, Increased H3K4me3 and MLL1 Amounts in the HPK1 Promoter Region, and Inhibited Tfh Cell Activity

**Figure 4:**
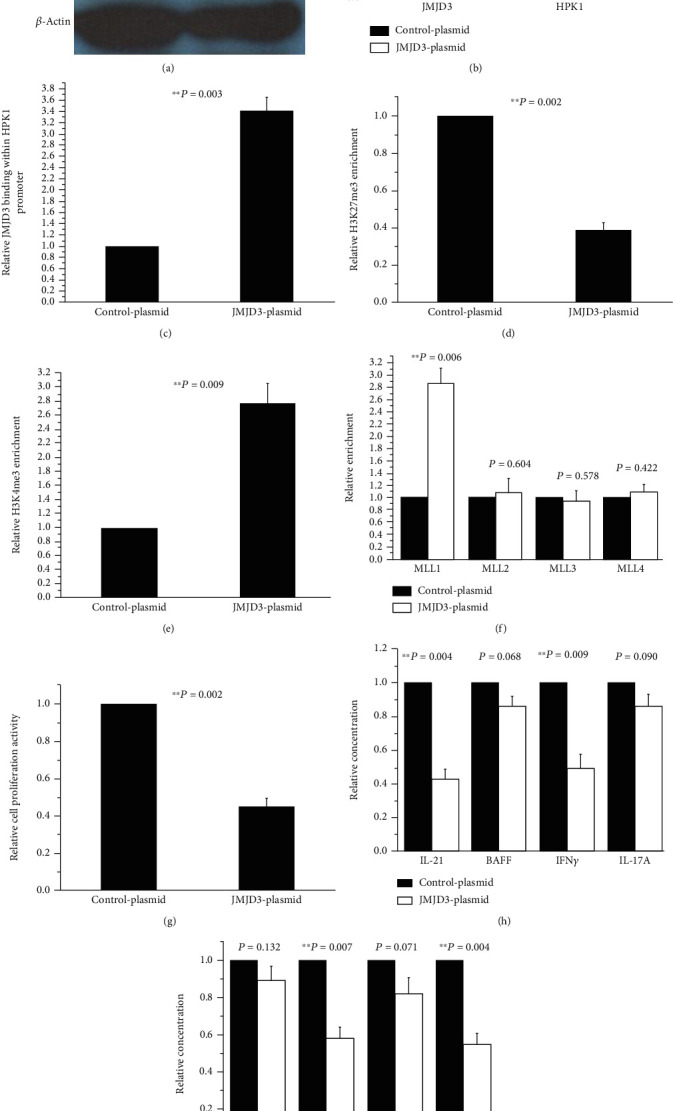
Effects of JMJD3 upregulation on Tfh cells from 3 SLE patients. (a, b) Relative JMJD3 and HPK1 protein levels in SLE Tfh cells transfected with pcDNA3.1 blank plasmid or pcDNA3.1-JMJD3-expressing plasmid were measured by western blot analysis after stimulation. *β*-Actin was used as endogenous control. (c–f) Relative JMJD3 (c), H3K27me3 (d), H3K4me3 (e), MLL1, MLL2, MLL3, and MLL4 (f) enrichments at the HPK1 promoter in SLE Tfh cells transfected with pcDNA3.1 blank plasmid or pcDNA3.1-JMJD3-expressing plasmid were quantified by combining ChIP and qPCR assays after stimulation. Results were normalized to input DNA (total chromatin). (g) The proliferation activity of SLE Tfh cells transfected with pcDNA3.1 blank plasmid or pcDNA3.1-JMJD3-expressing plasmid was assessed by MTT assays after stimulation. (h) The relative concentrations of IL-21, BAFF, IFN*γ*, and IL-17A in the supernatants of SLE Tfh cells transfected with pcDNA3.1 blank plasmid or pcDNA3.1-JMJD3-expressing plasmid were examined using ELISA after stimulation. (i) After stimulation, some SLE Tfh cells transfected with pcDNA3.1 blank plasmid or pcDNA3.1-JMJD3-expressing plasmid were cocultured with autologous B cells for 8 days, whereafter the concentrations of IgM, IgG1, IgG2, and IgG3 in the supernatants were detected by ELISA. The data from every pcDNA3.1 blank plasmid group member were set as 1, and the multiple of every pcDNA3.1-JMJD3-expressing plasmid group member relative to its homologous pcDNA3.1 blank plasmid group member was calculated.

Whereafter, we transfected Tfh cells from three SLE patients with pcDNA3.1 blank plasmid or pcDNA3.1-JMJD3-expressing plasmid. Expectedly, after 72 h of transfection, the protein levels of JMJD3 and HPK1 (Figures 4(a) and 4(b)) and the enrichments of JMJD3 (Figure 4(c)) and H3K4me3 (Figure 4(e)) at the HPK1 promoter all elevated greatly in SLE Tfh cells transfected with the JMJD3-overexpressing plasmid. The abundance of H3K27me3 lowered markedly (Figure 4(d)), and MLL1 binding within the HPK1 promoter region increased obviously (Figure 4(f)), while there were no significant differences in abundances of MLL2, MLL3, and MLL4 between the two groups (Figure 4(f)). Meanwhile, the proliferation activities of Tfh cells decreased sharply (Figure 4(g)), the concentrations of IL-21, IFN*γ*, IgG1, and IgG3 attenuated noticeably (Figures 4(h) and 4(i)), but BAFF, IL-17A, IgM, and IgG2 did not change dramatically in the JMJD3-overexpressing plasmid group (Figures 4(h) and 4(i)).

### 3.5. H3K4me3 and MLL1 Enrichments at HPK1 Promoter in SLE Tfh Cells Diminished and Were Positively Correlated with JMJD3 Binding in This Region

**Figure 5:**
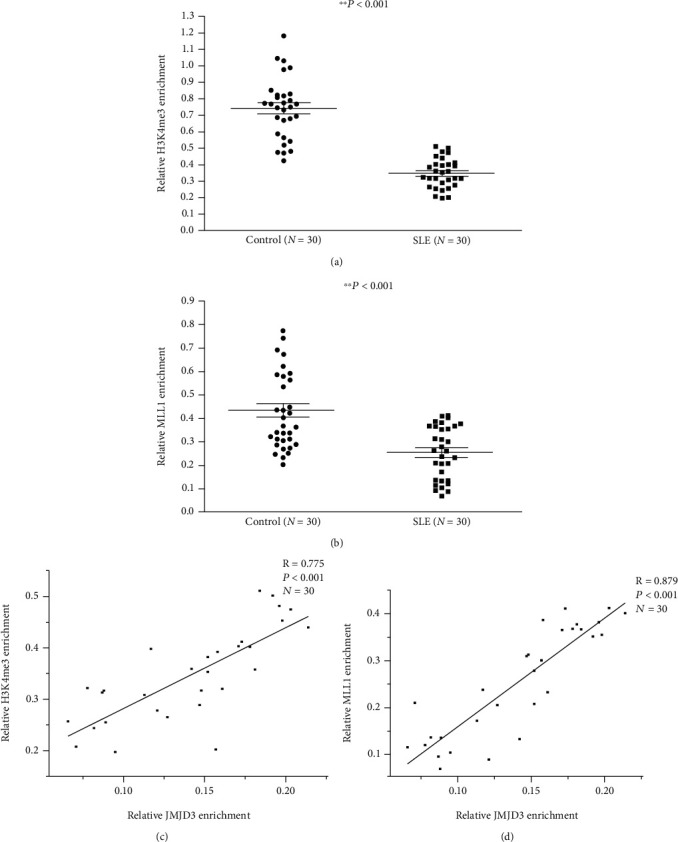
H3K4me3 and MLL1 enrichments at the HPK1 promoter in healthy and SLE Tfh cells. (a, b) Relative H3K4me3 (a) and MLL1 (b) enrichments at the HPK1 promoter in healthy and SLE Tfh cells were measured by combining ChIP and qPCR assays. Results were normalized to input DNA (total chromatin). (c, d) The correlations between H3K4me3 enrichment (c), MLL1 enrichment (d), and JMJD3 binding in SLE Tfh cells.

According to the transfection results, the levels of H3K4me3 and MLL1 at the HPK1 promoter in Tfh cells from the aforementioned SLE and control groups were detected by combining ChIP and qPCR assays. The results showed that H3K4me3 (Figure 5(a)) and MLL1 (Figure 5(b)) enrichments at the promoter in Tfh cells of the SLE group were reduced compared with those in the control group, and JMJD3 binding was positively correlated with H3K4me3 (Figure 5(c)) and MLL1 (Figure 5(d)) levels in Tfh cells from the SLE group.

## 4. Discussion

Due to its role in immune regulation, HPK1 has been found to play an important role in various autoimmune diseases, tumors, and inflammatory diseases. Our previous study also confirmed that underexpression of HPK1 led to the excessive activation of Tfh cells in SLE patients, resulting in the intensified inflammatory response [17]. In order to explore the reasons for the reduced HPK1 in SLE Tfh cells, the H3K27me3 enrichment in HPK1 promoter region was detected, and it was found that the H3K27me3 number in this region of Tfh cells from SLE patients increased strikingly, and it was negatively correlated with the mRNA and protein expressions of HPK1. These results suggest that the elevated H3K27me3 level at the promoter leads to the inhibition of HPK1 in Tfh cells of SLE patients.

H3K27me3 amount is regulated by both histone methyltransferase and histone demethylase. The increased H3K27me3 enrichment at the HPK1 promoter in SLE Tfh cells suggested that the levels of these enzymes may be altered. Therefore, we further studied EZH2, JMJD3, and UTX bindings at HPK1 promoter in the two groups. The results showed that JMJD3 number at the HPK1 promoter region in SLE Tfh cells was reduced, and it was negatively correlated with H3K27me3 enrichment, while it positively correlated with HPK1 expression. There were no significant differences in EZH2 and UTX levels between the two groups. These results indicate that the decreased JMJD3 binding in the promoter region contributes to upregulated H3K27me3 level and ultimately inhibits HPK1 expression in SLE Tfh cells.

In order to further confirm the regulatory effects of JMJD3 on HPK1, JMJD3-siRNA was used to downregulate JMJD3 level in Tfh cells from healthy donors. The results revealed that JMJD3 binding at HPK1 promoter alleviated, H3K27me3 enrichment in the region upregulated, and HPK1 expression was profoundly lowered in the JMJD3-siRNA group. Concordantly, the use of JMJD3-overexpressed plasmid to increase JMJD3 amount in SLE Tfh cells produced opposite results. The data suggest that JMJD3 regulates HPK1 expression in Tfh cells, and this regulation is achieved at least in part by altering JMJD3 and H3K27me3 levels in the HPK1 promoter region.

Will the change in JMJD3 abundance affect HPK1 quantity by other way and regulate Tfh cell activity at last? We observed that H3K4me3 and MLL1 levels were reduced after downregulating JMJD3 at HPK1 promoter in Tfh cells from healthy controls. Meanwhile, the Tfh cells proliferated greatly, secreted more IL-21 and IFN*γ*, and stimulated autologous B cells to produce more IgG1 and IgG3. However, overexpression of JMJD3 at HPK1 promoter in SLE Tfh cells had the opposite effects. These results suggest that the attenuate JMJD3 binding at the HPK1 promoter in SLE Tfh cells not only elevates H3K27me3 number but also blocks the recruitment of MLL1, leading to downmodulated H3K4me3 enrichment. All these may further lead to inhibited HPK1 expression and result in Tfh cell overactivation ultimately. As the transfection not only altered JMJD3 binding at the HPK1 promoter but also changed overall JMJD3 expression, we cannot rule out the possibility that JMJD3 can also regulate the levels of MLL1, H3K4me3, HPK1, and Tfh activity through other pathways.

According to the transfection results, we detected H3K4me3 and MLL1 levels at the HPK1 promoter region in Tfh cells from the aforementioned SLE and control groups. The results indicated that MLL1 and H3K4me3 enrichments at HPK1 promoter in SLE Tfh cells were sharply alleviated, and JMJD3 binding at this region was positively correlated with MLL1 and H3K4me3 abundance. These results reconfirm that JMJD3 at the HPK1 promoter in Tfh cells of SLE patients may regulate MLL1 and H3K4me3 numbers, thereby influencing HPK1 expression and Tfh cell activity.

## 5. Conclusions

In summary, our results show that the decreased JMJD3 at the HPK1 promoter in Tfh cells from SLE patients leads to upregulation of H3K27me3 in this region and inhibits recruitment of MLL1, thereby downregulating H3K4me3 enrichment. These factors lead to HPK1 underexpression and Tfh cell overactivation and promote the onset and development of SLE in the end. Our findings reveal the epigenetic mechanisms of regulating HPK1 expression in SLE Tfh cells and provide new ideas for the treatment of SLE.

## Figures and Tables

**Table 1:** Profiles of SLE patients.

Patient	Gender	Age (years)	Disease duration (months)	SLEDAI	Medications
1	Female	25	8	6	Pred 35 mg/d, MMF 1.5 g/d
2	Female	26	4	4	Pred 30 mg/d
3	Female	25	5	10	Pred 40 mg/d, CsA 150 mg/d, TG 30 mg/d
4	Female	30	3	8	None
5	Female	34	6	8	Pred 40 mg/d, HCQ 0.2 g/d
6	Male	32	25	4	HCQ 0.2 g/d
7	Female	36	23	6	Pred 30 mg/d, HCQ 0.2 g/d
8	Female	27	7	10	Pred 40 mg/d, MMF 1.5 g/d
9	Female	25	32	16	Pred 40 mg/d, HCQ 0.2 g/d
10	Female	22	6	20	Pred 50 mg/d, MMF 1.5 g/d, HCQ 0.2 g/d
11	Female	20	4	2	None
12	Female	21	8	0	Pred 15 mg/d
13	Female	28	10	2	Pred 10 mg/d, TG 30 mg/d
14	Female	33	13	1	Pred 20 mg/d
15	Female	32	11	6	Pred 25 mg/d, CsA 150 mg/d
16	Female	41	9	8	None
17	Male	40	3	6	Pred 40 mg/d, HCQ 0.2 g/d
18	Female	32	8	6	Pred 30 mg/d, CsA 150 mg/d
19	Female	35	21	0	Pred 5 mg/d
20	Female	24	14	6	None
21	Female	26	6	8	Pred 40 mg/d, MMF 1.5 g/d
22	Male	32	9	6	None
23	Female	35	7	2	TG 30 mg/d
24	Female	22	15	0	Pred 10 mg/d, HCQ 0.2 g/d
25	Female	33	10	7	Pred 35 mg/d
26	Female	35	4	8	Pred 35 mg/d, HCQ 0.2 g/d
27	Female	27	21	8	Pred 35 mg/d, MMF 1.5 g/d
28	Female	25	2	4	None
29	Female	22	9	2	Pred 15 mg/d, HCQ 0.2 g/d
30	Female	23	6	16	Pred 40 mg/d, CsA 150 mg/d, HCQ 0.2 g/d

Abbreviations: SLE: systemic lupus erythematosus; SLEDAI: systemic lupus erythematosus disease activity index; Pred: prednisone; MMF: mycophenolate mofetil; CsA: cyclosporin A; TG: tripterygium glycoside; HCQ: hydroxychloroquine.

**Table 2:** Profiles of healthy controls.

Healthy control	Gender	Age (years)
1	Female	24
2	Female	22
3	Male	27
4	Female	28
5	Female	27
6	Female	35
7	Female	32
8	Female	36
9	Female	22
10	Female	21
11	Female	24
12	Female	32
13	Female	24
14	Male	26
15	Female	41
16	Female	28
17	Female	33
18	Female	28
19	Female	27
20	Female	40
21	Female	31
22	Female	25
23	Female	24
24	Female	23
25	Female	25
26	Male	28
27	Female	34
28	Female	20
29	Female	21
30	Female	26

## Data Availability

All data generated or analyzed during this study are included in this article. Further enquiries can be directed to the corresponding author on reasonable request.
